# Investigates the ability of plant extracts from *Lens culinaris* to protect zucchini from the *Zucchini yellow mosaic virus* (ZYMV)

**DOI:** 10.1038/s41598-024-62128-6

**Published:** 2024-05-28

**Authors:** Rania Ali, Gamal Eldidamony, Ahmed Askora, Abdelmoneim Galal

**Affiliations:** Botany and Microbiology Department, Faculty of Science, Zagzig University, Zagazig, 82524 Egypt

**Keywords:** Metabolism, Peroxidase, Pigment, Lentil lectin, *Zucchini yellow mosaic virus*, Microbiology, Plant sciences

## Abstract

Evaluate the impact of extracts from the *Lens culinaris* plant on a number of physiological and biochemical parameters in squash leaves infected with ZYMV in this work. Compared to the untreated leaves, ZYMV infected leaves showed a range of symptoms, such as severe mosaic, size reduction, stunting, and deformation. Analysis of physiological data revealed that *L. culinaris* extract lectin therapies and viral infections had an impact on metabolism. Protein, carbohydrate, and pigment levels were all lowered by viral infection. However, phenolic compounds, total protein, total carbohydrates, total amino acids, proline, total chlorophyll and peroxidases levels are considerably elevated with all extract therapies. The other biochemical parameters also displayed a variety of changes. Moreover shoot length, number of leaves and number of flowers was significantly increased compared to viral control in all treatments. The *L. culinaris* extract treatment increases the plant’s ZYMV resistance. This is detectable through reduction of the plants treated with lentil lectin pre and post virus inoculation, reduction in disease severity and viral concentration, and percentage of the infected plants has a virus. All findings demonstrate significant metabolic alterations brought by viral infections or *L. culinaris* extract treatments, and they also suggest that exogenous extract treatments is essential for activating the body’s defences against ZYMV infection.

## Introduction

The vegetable zucchini (*Cucurbita pepo*, L.) is a popular crop in Egypt and many other African countries. It is recognised as an essential food cucurbit crop for human nutrition in developing countries^1^. Cucurbit plant infections caused by a variety of viruses have the potential to cause serious problems all over the world. Fruits on virus-infected cucurbita plants are deformed and yield is reduced^2^. The ZYMV virus, a member of the Potyvirus genus, is one of the most economically significant viruses on cucurbit crops in the world^3,4^. Melon (*Cucumis melo*), cucumber (*C. sativus*), summer squash (*C. pepo*), pumpkin (C. maxima), winter squash (*C. moschata*), and watermelon (*Citrullus lanatus*) are crops that are important to the economy are vulnerable to ZYMV. Serious mosaic, stunting, yellowing, necrosis, loss of leaf area, deformed leaves and fruits, and notable output decreases are common ZYMV symptoms^5^. Since its discovery in Italy in 1981^6^, ZYMV has been discovered in Asia, Africa, America, and other continents.

Cucurbit crops and other indicator plants may exhibit a range of biological features associated with ZYMV^7^. ZYMV belongs to the genus Potyvirus and the family Potyviridae, according to Asad and Ashfaq^8^. Its RNA genome, which consists of 9600 nucleotides, is contained within flexible filamentous particles with a diameter of 11 nm and a length of 750 nm. At the 3 nm end of the polyprotein, which codes for ten, the viral protein is covalently attached to the poly (A) tail^9^. Using a range of methods, ZYMV in squash and other cucurbit crops was identified and described. Early removal of contaminated plants and the application of certain insecticides to manage vectors were the principal techniques employed to fight plant virus disease, however these techniques were unable to stop viruses carried by aphids in a non-persistent way. Therefore, the goal of the research was to identify chemicals that, by employing certain plant extracts to induce systemic resistance in plants, were more efficient at treating plant virus illnesses^10^. Plants’ natural defences, such as systemic acquired resistance (SAR), could be induced to manage viral diseases. A type of induced resistance known as “systemic acquired resistance” is triggered in a plant when it is subjected to elicitors from artificial, virulent, or non-pathogenic microorganisms. When Ray and colleagues studied Botrytis cinerea in 1901, they recognised for the first time the natural phenomena of resistance in response to pathogen infection or plant immunity^11^. Although it has been seen, the phenomena of SAR against disease in plants after infection was often unreported as early as the nineteenth century. When the lower leaves of Dianthus barbatus were injected with the Carnation mosaic virus (CarMV), the higher leaves looked resistant to the infection, a phenomenon first reported in 1952 by Gilpatrick and Weintraub. By inducing the defence mechanism in vulnerable hosts, the glycoprotein promotes the plant system to produce new proteins^12^, and it prevents viral infection by impeding virus replication^13^. Strong antiviral activity was found in the sap that was extracted from the leaves of host plants sprayed with *Boerhaavia diffusa* glycoprotein^14^. On the other hand, Waziri^10^ found that no similar activity was seen in the sap of the untreated control plants. The explanation states that antiviral agent [AVA] protein, which is lacking in control plants, is present in the sap of treated leaves^10^. It has been documented that *B. diffusa* glycoprotein effectively induces high systemic resistance in several vulnerable hosts via boosting immunological system^15^. The effects of *Lens culinaris*, Glycine max extract, and ZYMV infection are investigated in this study. treatments with SA V on a number of physiological and biochemical traits in pumpkin (*C. pepo*) leaves. In particular, protein composition, POX activity, carbohydrate content, and other component analyses, as well as SDS-PAGE results, demonstrate the emergence of resistance to extract treatments for ZYMV infection.

## Materials and methods

Experimental research and field studies on cultivated plants, including the collection of plant material, according to institutional guidelines and legislation.

### Source of ZYMV virus and propagation

A total of 150 naturally infected leaves and knobbed fruits of squash (*C. pepo*) samples showing clear mosaic, yellowing symptoms were collected from fields in Sharkia Governorate, Egypt. The presence of ZYMV was checked in the collected samples by serological means using DAS-ELISA technique according to^16^, using anti-ZYMV polyclonal antibodies (Agdia, Elkhart, India). The absorbance of ELISA reactions at 405 nm was measured, and twice of the absorbance values of healthy plants were considered positive. ZYMV was mechanically transmitted into (*Chenopodium amaranticolor* plants), then propagated and maintained in squash plants (*C. pepo* L.). The symptoms were checked 3 weeks after inoculation (DPI), and the infected leaves were frozen and used as an inoculum source in subsequent studies**.** All experiment was carried out in the greenhouse of Botany and Microbiology Department, Faculty of Science, Zagazig University, Cairo, Egypt.

### Electron microscopy

Morphological analysis of the isolated ZYMV was examined by transmission electron microscopy. A drop of purified ZYMV was placed on 200 mesh copper grids with carbon-coat formvar films and the excess was drawn off with filter paper. A saturated solution of 2% uranyl acetate was then placed on the grids and excess was drawn off. Specimens were examined with an electron microscope Hitachi H600A of the Faculty at Agriculture, Mansoura University.

### Host range

For host range evaluation, the isolated ZYMV was mechanically transmitted to different plants of *C. pepo*, *C. moschata*, *Cucumis sativus*, *C. melo*, *C. lanatus*, Luffa *acutangula*, *C. amaranticolor*, and other belonging to Leguminaceae, *Solanaceae, Asteraceae, Graminaceae,* *Chenopodiaceae*. Plants were sown in a mixture of loamy soil in pots (30 cm diameter). The pots were placed in the green house of the Faculty of Science, Zagazig University at 24–30 °C and kept at a constant relative water content of 100%. The cotyledons of plants with fully expanded first true leaves (five for each species) were dusted with carborundum powder and then mechanically inoculated using crude extracts of ZYMV ELISA-positive leaf material in 0.01 M phosphate buffer (pH 7.2). Plants were observed for symptoms over a 5-week period. Plants used in the host range studies were grown in pots and maintained at 18–25 °C in an insect-proof greenhouse.

### Molecular identification

Using the RNeasy_Plant Mini Kit (Qiagen), total RNAs were extracted from the leaves of the original infected hosts to prevent mutations from accumulating during transfer to and propagation in experimental hosts. The ZYMV P1 gene’s forward and reverse primers were utilised to amplify the particular genomic region. There were two steps in the RT-PCR process. Reverse transcription of whole RNA employing a revers primer produced the first strand cDNA. 1 µl of total RNA sample, 1 µl of reverse primer, 9 µl of water, 2 µl of 10 mµ NTPs, 2 µl reverse transcriptase enzyme (M.Mluv), and 2 µl of 10X M.Mluv buffer are all added to the experiment. After gently mixing the mixture, centrifuging it, and letting it sit at 37 °C for 60 min, the reaction was halted by raising the temperature to Five minutes at 70 °C. For the P1 gene of the virus, PCR amplification was carried out using the following primers: (5′-AGTGGCACCTGGCCACATGGC-3′) and (3′-CATCTCAGTGTGCCGCATTCG-5′) as the forward and reverse primers, respectively.

Additionally, the following cycling parameters were used (Techne Genius, Merck): 5 min of initial denaturation at 94 °C, 35 cycles of 94 °C/1 min, 54_C/45 s, 72_C/1 min, and 10 min of final extension at 72 °C. Using a Mega BACETM 1000 DNA Analysis System (Amersham Biosciences) and gel-purified PCR products (QIAexII gel extraction kit, Qiagen), the sequencing reaction was primed using the same oligonucleotides as were used for PCR^17^.

### Plant lectin extracts

Dry seeds of *L. culinaris and Glycine max* plants were collected from local market; seeds were ground to a powder in electric mill and filtered through 50 mesh grit. The powder was defatted with Hexane (1:5 w/v ratios) for 30 min, and then the process was repeated until the hexane was no longer mixed with the fat. Hexane was further eliminated using the volatilization of dry seed powder at ambient temperature. The dry powder was combined with a 0.02 M phosphate buffer solution with a pH of 7.2 (1:5 w/v) and left overnight at 4 °C with continuous stirring using a shaker. Immediately after, the mixture was filtered through a multi-layer cheesecloth filter. Centrifuging the obtained solution took place at 4 °C for 20 min at 10.000 rpm. The precipitation residue was removed, and the supernatant solution was kept at 4 °C for further use. Protein precipitation by ammonium sulphate: was completed using a precipitant of 90% ammonium sulphate saturation, and after an hour of incubation, then centrifuging the mixture for 30 min at 10,000 rpm. A sufficient amount of buffer was used to dissolve the precipitated pellet, which was then collected for further use^18^. Then the pellet which was dissolved in phosphate buffer (pH 7.2) dialyzed overnight in the same buffer at 4 °C. The produced samples from the dialysis process were considered partially purified or dialyzed lectins.

#### Protein concentration and separation

The lectin protein concentration was measured by applying the Bradford^19^ method, which employed bovine serum albumin (BSA). Ten percent acrylamide gels were used for SDS PAGE in compliance with Laemmli’s technique^20^. Forty milligrammes of protein samples in an equivalent volume were added to a buffer containing 0.125 M TriseHCl, pH 6.8, 4% SDS, 20% glycerol, 10% 2-mercaptoethanol, and bromophenol blue as a tracking dye. The resulting mixture was heated in a water bath for three minutes at 96 C before being loaded onto gel for 6 h at 10 C using a run buffer that contained 0.025 M Tris, 0.192 M glycine, and 0.1 percent SDS. Using the blue dye Commassie Brilliant, there were visible protein bands.

#### Haemagglutination activation test

The experiment was carried out in 96-well plates. 50 μl of 4% (w/v) RBCs were mixed with two-fold serial dilutions of the examined lectin samples in 5 mM phosphate buffer saline (pH 7.2). The mixes were then incubated for an hour at 37 °C. A reference (50 µl of 4% cell suspension and 50 µl of PBS in place of protein solution)^21^. The titer, which is the reciprocal of the lowest protein concentration at which erythrocyte agglutination could be seen, was used to express the haemagglutination activity.

#### Haemagglutination inhibition test

In phosphate-buffered saline, a series of two-fold dilutions of the following sugars were prepared: galactose, glucose, glucose, mannose, fructose, and sucrose. Every dilution of the sugars under test was combined with an equivalent amount of 50 μl of lectin solutions that were isolated from two leguminous seeds and showed positive haemagglutination activity. After an hour of room temperature incubation, 50 μl of a 4% human red blood cell suspension was added to the mixes^21^. 50 μl of protein solution and 50 μl of 4% red blood cells made up the negative control. It was possible to describe the suppression of haemagglutination as positive or negative.

### lectin treatments and virus inoculation

After 10 days of growth, zucchini plants were divided into groups for treatment:

#### Post inoculation experiment (treatment after virus infection)

After dusting the leaves with carborundum, the cotyledonary leaves of *C. pepo* L. plants were inoculated with a virus inoculum (100 μl/leaf). The inoculated leaves were then rinsed with distilled water. Following a virus inoculation period of 6, 12, 24, and 48 h, 100 μl of plant extract filtrate per leaf was applied to the leaves. After 21 days, the emerging symptoms were noted, and the percentages of inhibition were computed. The number of leaves, length of the stalk, and quantity of blooms were counted. Both the general control and the viral control were computed^22^.

#### Pre inoculation experiment (treatment before virus infection)

Before 6, 12, 24 and 48 h. of virus inoculation, the leaves were treated with plant extract filtrate 100 μl/leaf. The cotyledonary of squash were inoculated with virus (100 μl/leaf) after dusting the leaves with carborundum, then the inoculated leaves were washed with distilled water. The developing symptoms were recorded after 21 days and the percentages of inhibition were calculated. Number of leaves, shoot length and Number of flowers were determined^23^.

#### Seed soaking treatment

After being treated in plant extract filtrate for 2, 4, or 6 h, the squash seeds were planted in pots. Following germination, 100 μl of ZYMV was injected into the cotyledonary leaves. Up to 21 days were spent observing and documenting the symptoms. It was calculated what the inhibition percentages were. For each phase, seeds were soaked in distilled water to ensure they were healthy control seeds. Squash seeds are subjected to a distilled water soak, allowed to sprout, and then mechanically injected with a single virus strain. According to Gondim et al.^24^, the number of leaves, shoot length, and flowers were counted.

### Determination of proline content

The approach given by Sadasivam and Manickam^25^ is used to estimate proline. Homogenizing 0.1 gm of plant material in 2 ml of 3% aqueous sulphosalicylic acid yields the extraction. The homogenate should be filtered using Whatman No. 2 filter paper. Add 2 ml of filterate, 2 ml of glacial acetic acid, and 2 ml of acid ninhydrin to a test tube (prepared by warming 1.25 gm ninhydrin in 30 ml glacial acetic acid and 20 ml 6 M phosphoric acid with agitation until dissolved). For 1 h, heat it in a pot of boiling water. Put the tube in an ice bath to stop the reaction. Stir thoroughly for 20–30 s after adding 4 ml of toluene to the reaction mixture. The toluene layer should be separated and warmed to room temperature.

### Determination of carbohydrates and total free amino acid contents

The phenol–sulphuric acid reaction of the sample's acid extract allowed for the estimation of total carbs. 100 mg of the plant sample should be weighed into a boiling tube. After adding 10 ml of 2.5 N HCl, hydrolyze it by holding it in a boiling water bath for 3 h before cooling at room temperature. Use sodium carbonate to neutralise it until effervescence. For analysis, centrifuge the sample and gather the supernatant^25^. To 0.5 ml of phenol (20% w/v) in a colorimetric tube, 100 µl of the extract were added. After that, 5 cc of concentrated sulfuric acid was quickly added while being shaken. The tubes were shaken and placed in a water bath at a temperature of 25–30 for 10–20 min after standing for 10 min. In replacement of the sugar solution, distilled water was used to create the blanks. At 490 nm, a blank surface is used to test the yellow-orange color’s absorption. g of glucose per gramme of fresh weight is how total carbohydrates are stated. The method described by used ninhydrin reagent to colorimetrically measure total amino acids. A 1 ml sample and 1.9 ml mixture of ninhydrin, citrate buffer, and glycerol are included in the reaction mixture. This combination is made up of 0.5 ml of a 1 percent ninhydrin solution in 0.5 M citrate buffer (PH 5.5), 0.2 ml of 0.5 M citrate buffer (PH 5.5), and 1.2 ml of glycerol. The mixture was warmed in a boiling water bath for 10 min before cooling in a bath of running water. At 570 nm, the developing colour was read. In terms of ug alanine per gm of body weight, the amino acids were expressed. Protein reagent was made by dissolving 100 mg of Coomassie Brilliant blue G-250 in 50 ml of 95 percent ethanol for the purpose of determining the total amount of soluble proteins. 100 ml of this solution at 85% (W/V) phosphoric acid were added to this solution. A final volume of 1 L was achieved by diluting the obtained solution. 50 mL of the sample solution or 50 ml of serial concentrations containing 10–100 g of bovine serum albumin were pipetted into test tubes to create the standard curve. Phosphate buffer was used to adjust the test tube's volume to 1 ml (0.1 M,pH 6.6). Test tubes containing five millimetres of protein reagent were filled with the substance, which was then mixed either by inversion or vortexing. After two minutes and before 1 h, the absorbance at 595 nm was measured against a blank made from 1 ml of phosphate buffer and 5 ml of protein reagent.

### Determination of total phenolics

Using the modified Folin-Ciocateu method, the total amount of phenolics in the extracts was calculated^26^. The Folin–Ciocalteu reagent was made by mixing 700 ml of deionized water with 100 gm sodium tungstate, 25 gm phosphomolybdic acid, 100 ml HCl, and 50 ml orhphosphoic acid (85%). After being refluxed for 10 h, the flask was cooled, and 150 g of lithium sulphate was then added. The solution was given a few drops of liquid bromine to turn it yellow, then deionized water was used to bring the total volume to 1 l. Test tubes were filled with 200 µl of plant extracts, 1 millilitre of Folin–Ciocalteu reagent, and 0.8 millilitres of sodium carbonate (7.5%). After mixing, the tubes were let to stand for 30 min. Measurements of absorption at 760 nm were made against a blank that contained nothing but the sample. The amount of total phenolic content was calculated using gallic acid standard (5 gm%) and reported as mg of gallic acid for every gm of the original sample’s dry weight (mg GA/gdw).

#### Extraction and measurement of chlorophyll a, b and total chlorophyll (a + b)

Chlorophylls were extracted in 80% acetone, and the absorption coefficient can be used to calculate the number of chlorophylls present: In 1971, Witham et al. The leaves were dried for four days (45 C0) in an oven. One gramme of finely ground leaf powder was combined with five millilitres of 80 percent acetone and ground in a clean mortar. The supernatant was used to measure the amount of chlorophyll by centrifuging the mixture at 5000 rpm for five minutes. The absorbance of the solution at 663 nm for chlorophyll A, 645 nm for chlorophyll B, and 652 nm for total chlorophyll was then compared to the solvent blank. Witham et al.^27^ used the following equation to calculate the number of chlorophylls: In the following equation, factor = 20.2 for chlorophyll is found: mg chlorophyll/gdw = Factor × (A) × V/(1000xw). In order to determine the amount of chlorophyll per gramme of dry weight (mg/gdw), Witham et al.^27^ calculated the number of chlorophylls using the following equation: Factor = 20.2 for chlorophyll A, 8.02 for B, and 28.8 for total A = absorbance. V is the total volume of the chlorophyll extract. W is the weight of the extracted tissue.

#### Statistics

At a probability level of 0.05, all data were compared using the analysis of variance (ANOVA) and least significant difference (LSD) tests.

## Results

### ZYMV detection and propagation

**Figure 1:**
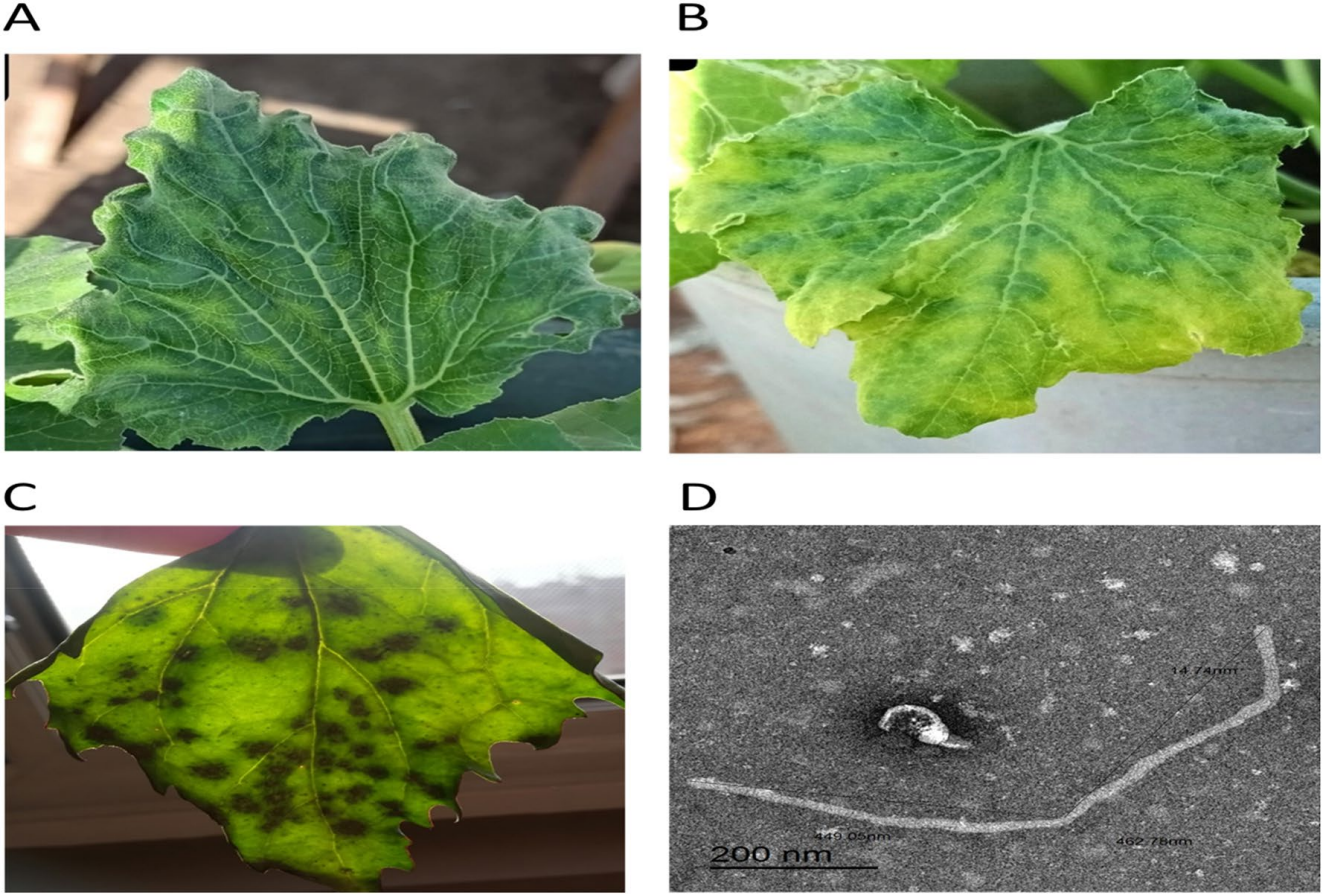
(**A** and **B**) External viral symptoms on *C. pepo* cv. Eskandarani (**C**) *Chenopodium murale* plants after mechanically inoculated with the isolated virus. (**D**) Electron micrograph showing flexible filamentous particles of ZYMV isolate negatively stained with phosphotungstic acid with magnification power 20000×.

A total of 150 samples exhibiting ZYMV-like symptoms (yellow mosaic, leaf deformation, severe blistering, and knobbed fruits), were screened by DAS-ELISA technique for ZYMV detection. ZYMV DAS-ELISA positive samples were collected and used as inoculum sources. One ZYMV isolates were chosen for further experiments. The virus isolate was propagated as a pure ZYMV source in propagation host *C. pepo* as systemic host and *Chenopodium murale* as local host. The infectious sap showed (severe mosaic, blisters, yellowing, mottling, crinkles, vein banding and leaves malformations) on zucchini leaves and (small round necrotic local lesions) on *C. murale* under greenhouse condition after 21, 7 days post inoculation respectively. The developed symptoms were observed on zucchini and *C. murale* leaves (Fig. 1A–C). ZYMV DAS-ELISA positive samples were collected and used as inoculum sources.

### Morphological characterization (transmission electron microscopy)

ZYMV virion was purified and examined under a transmission electron microscope. Transmission electron microscopy showed that ZYMV particles have a flexuous filament with length of about 750 nm and 11 nm wide in diameter after staining with phosphotungstic acid (PTA),PH (5–8) (Fig. 1D).

### Host range of ZYMV

**Figure 2:**
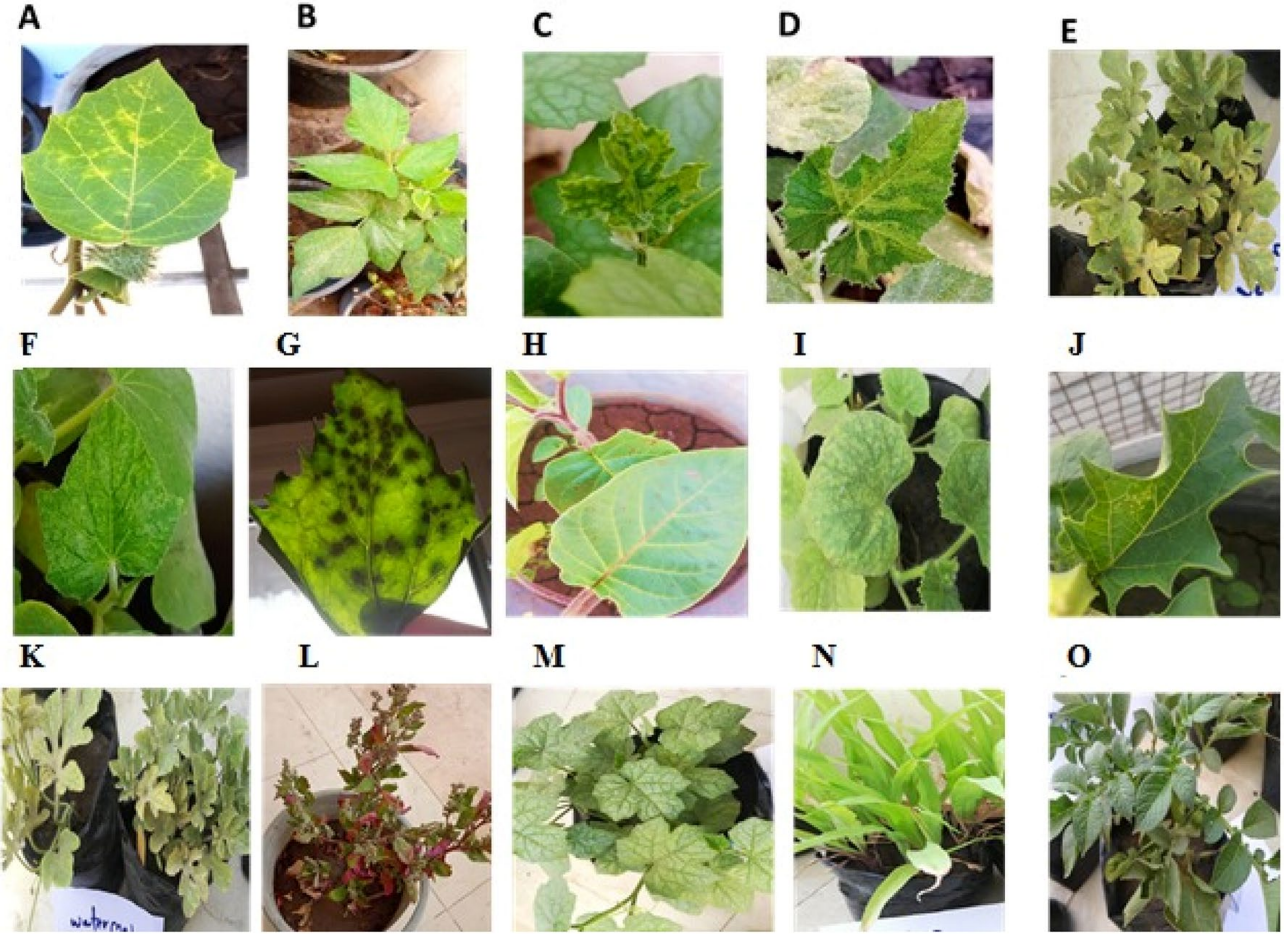
External symptoms appearing on some inoculated plants as a host range. (**A**) *Datura metal,* (chlorotic local lesions), (**B**) *Phaseolus vulgaris* cv. white Kidney. (Severe mottling)*,* (**C**) *Luffa acutangula* L., (*SM* severe mosaic, *LMf* leaf malformation, *VC* vein banding, *Mo* mottling, *T* tumors)*,* (**D**) *Cucurbita moschata* ‘Butternut, (*SM* severe mosaic, *SMo* severe mottling, *Y* yellowing, *LMf* leaf malformation)*,* (**E**) *Citrullus lanatus,* (*SM* severe mosaic, *Mo* mottling, *Y* yellowing, *VB* vein banding), (**F**) *Cucumis sativus* (*SM* severe mosaic, *Y* yellowing, *LMf* leaf malformation), (**G**) *Chenopodium album* (necrotic local lesions), (**H**) *Datura metal* (*necrotic local lesions*), (**I**) *Cucumis melo var. cantalupensis* (*YLL* yellowing, *SM* Severe mosaic, *Mo* mottling*,* (**J**) *Datura stromonium* *CLL* (chlorotic local lesions), (**K**) *Citrullus lanatus,* (**L**) *Chenopodium amaranticolor* *CLL* chlorotic local lesions (*M*) *Luffa acutangula L*., (**N**) *Zea mays* (*NS* No symptoms, (**O**) *Solanum tuberosum* (*LC* Leaf curling).

Twenty nine plant species were tested for susceptibility to ZYMV by sap-inoculation, most species belonging to the family cucurbitaceae developed systemic symptoms, *C. pepo*, *C. moschata*, *Cucumis melo*, *C. lanatus*, and *Luffa acutangula*. Species belong to Chenopodiaceae, *Chenopodium amaranticolor*, *Ch. Murale, Ch.quinoa,* one to Leguminaceae, *Vigna unguiculata cv.*and two to solanaceae, *Datura metal and Datura stromonium* were found susceptible and develop local symptoms Fig. 2.

#### Serological identification

**Figure 3:**
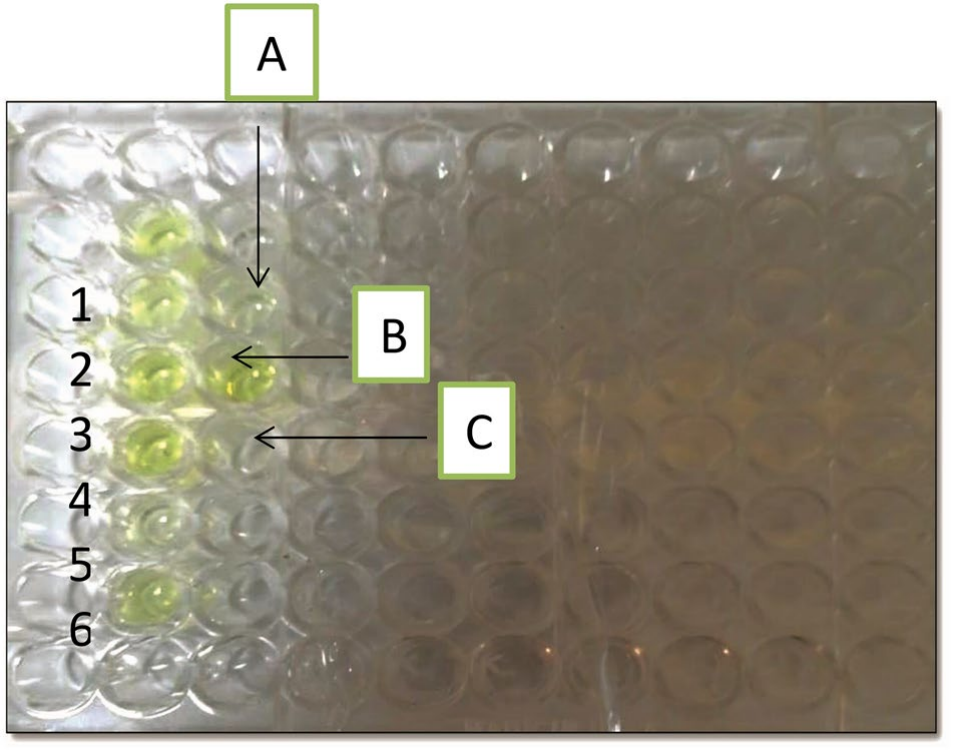
Serological detection of ZYMV naturally infected zucchini plants by (DAS-ELISA): (**A**) Blank, (**B**) Negative control, (**C**) Positive control, Numbers (1–6) refers to different samples infected with ZYMV.

*Double antibody sandwich ELISA (DAS-ELISA)*: The virus antigen was serologically precipitated against specific polyclonal IgG-ZYMV by (DAS-ELISA) assay Table 1. It was found that the Double antibody sandwich ELISA (DAS-ELISA) sensitive to detect ZYMV in all infected samples. A yellow color was developed with infected zucchini in the positive reaction, whereas extracts from healthy plants remain colorless in the negative reactions and Fig. 3.

**Table 1:** Serological detection of ZYMV naturally infected zucchini plants by (DAS-ELISA).

Blank	Negative control	Positive control	Sample1	Sample2	Sample3	Sample4	Sample5	Sample6
0.113	0.589	2.176	1.623	1.211	2.101	2.363	0.627	1.438

#### Molecular identification

**Figure 4:**
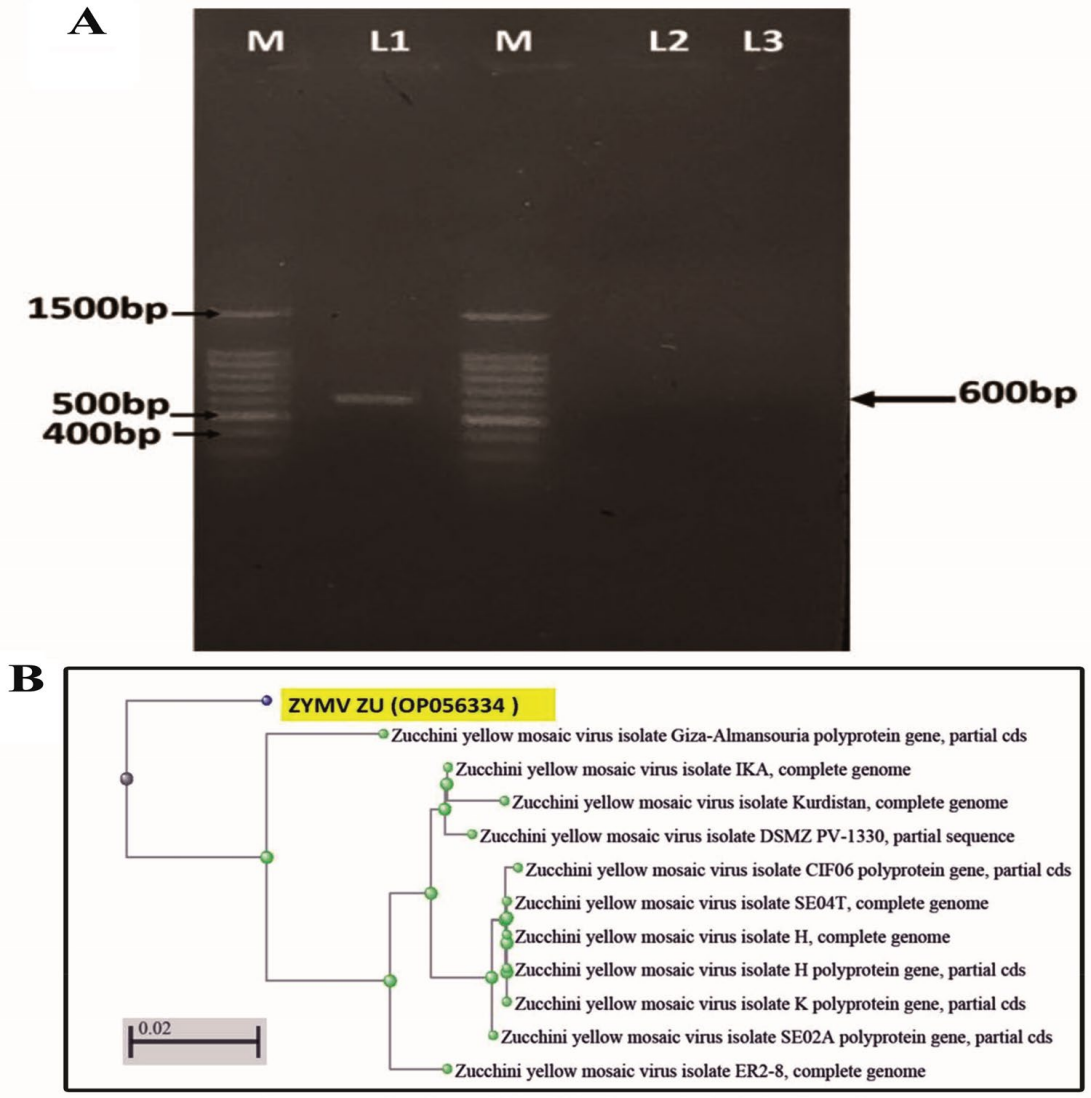
Agarose gel electrophoresis patterns of RT-PCR products of ZYMV isolate. M: 100 bp DNA ladder (Biomatik). L1: Sample of the naturally infected zucchini plant. L2 and L3: Negative zucchini plant (Healthy).

*(RT-PCR)*: RT-PCR was used for the detection of ZYMV coat protein (p1) gene in infected squash. PCR fragment of the expected size 600 bp was amplified. Electrophoresis of PCR amplicons is shown in Fig. 4. PCR- amplified fragment for the P1 (protease) gene of ZYMV isolate was subjected to sequencing to determine the relevance with other ZYMV isolates. The P1 sequences for the isolated ZYMV (accession number OP056334), were quite similar, with a nucleotide sequence similarity of 98% between several ZYMV previously isolated.

#### Protein concentration and separation

Determination of lectin concentration: The crude extracts of two leguminous seeds were precipitated with ammonium sulfate (90%) followed by overnight dialysis. The yield of the dialysed lectins was reported at 90% saturation for the tested seeds, and the highest concentration (22.4 mg/ml) of dialysed lectins was recorded in lentil Table 2.

**Table 2:** Hemagglutination activity of partially purified lectins (90% fraction) extracted from lentil and soyabean on human blood group (O) and the concentration of protein (mg∕ml).

Plant lectin	Total protein (mg/ml)	Haemglution activity titre (mg)	Specific hemagglutination activity (titer/mg)
Soyabean	19.8	0.5	39.6
lentil	22.4	0.25	89.6

Analysis of protein patterns of the extracted lectins by poly acrylamide gel electrophoresis:

After determination of lectin concentration in dialysed samples of lentil and soya bean, they were loaded on SDS-PAGE gels, and the electropherogram showed the patterns of the extracted lectins. The data obtained by using (SDS-PAGE) was expressed as Soya bean lectin has 130 kDa and lentil lectin 46 kDa Supplementary Fig. 5.

**Figure 5:**
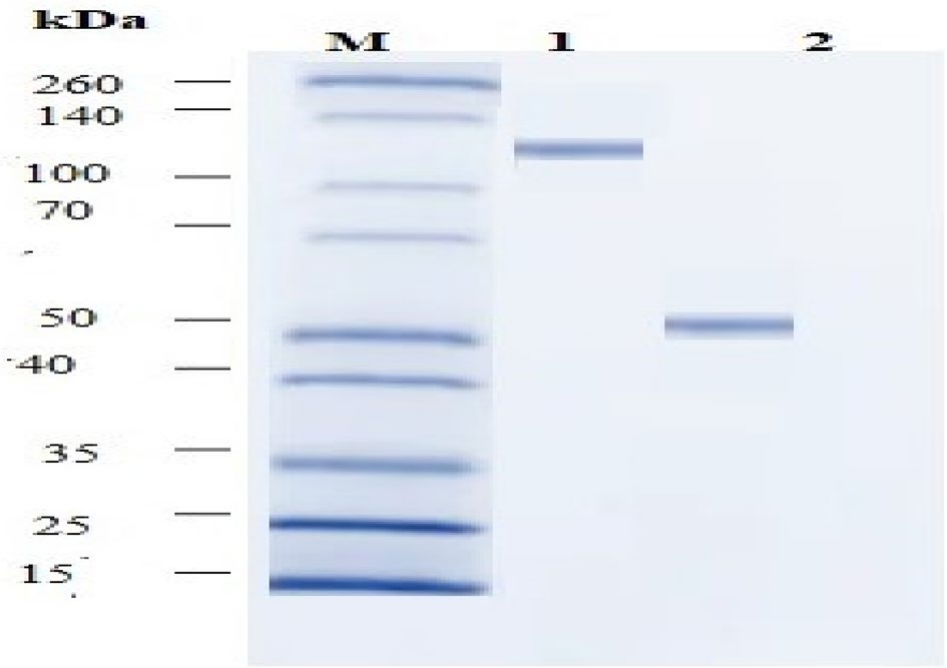
Electrophoresis of different concentrations of the two tested lectins (soya bean lectin and lentil lectin). Lane M: marker of the standard protein. Lane 1: soyabean lectin has 130 kDa. Lane 2: lentil lectin has lectin 46 kDa.

### Haemagglutination activation test

**Figure 6:**
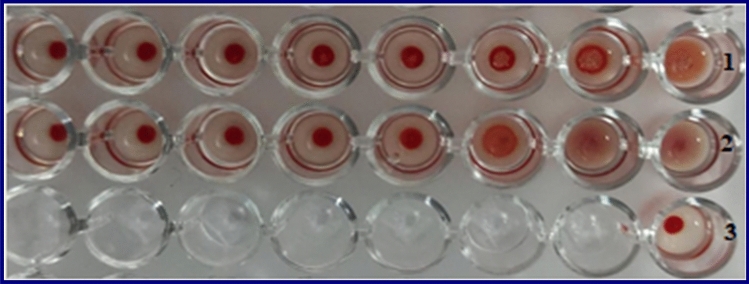
show the effect of different concentrations of lectins on the inhibition of human blood group (O). raw1 = soyabean lectin, raw2 = lentil lectin and raw3 = control (blood group only).

The different concentrations of dialysed lectin of lentil and soyabean agglutinated human blood group (O) shown in Fig. 6.

### Haemagglutination inhibition test

**Figure 7:**
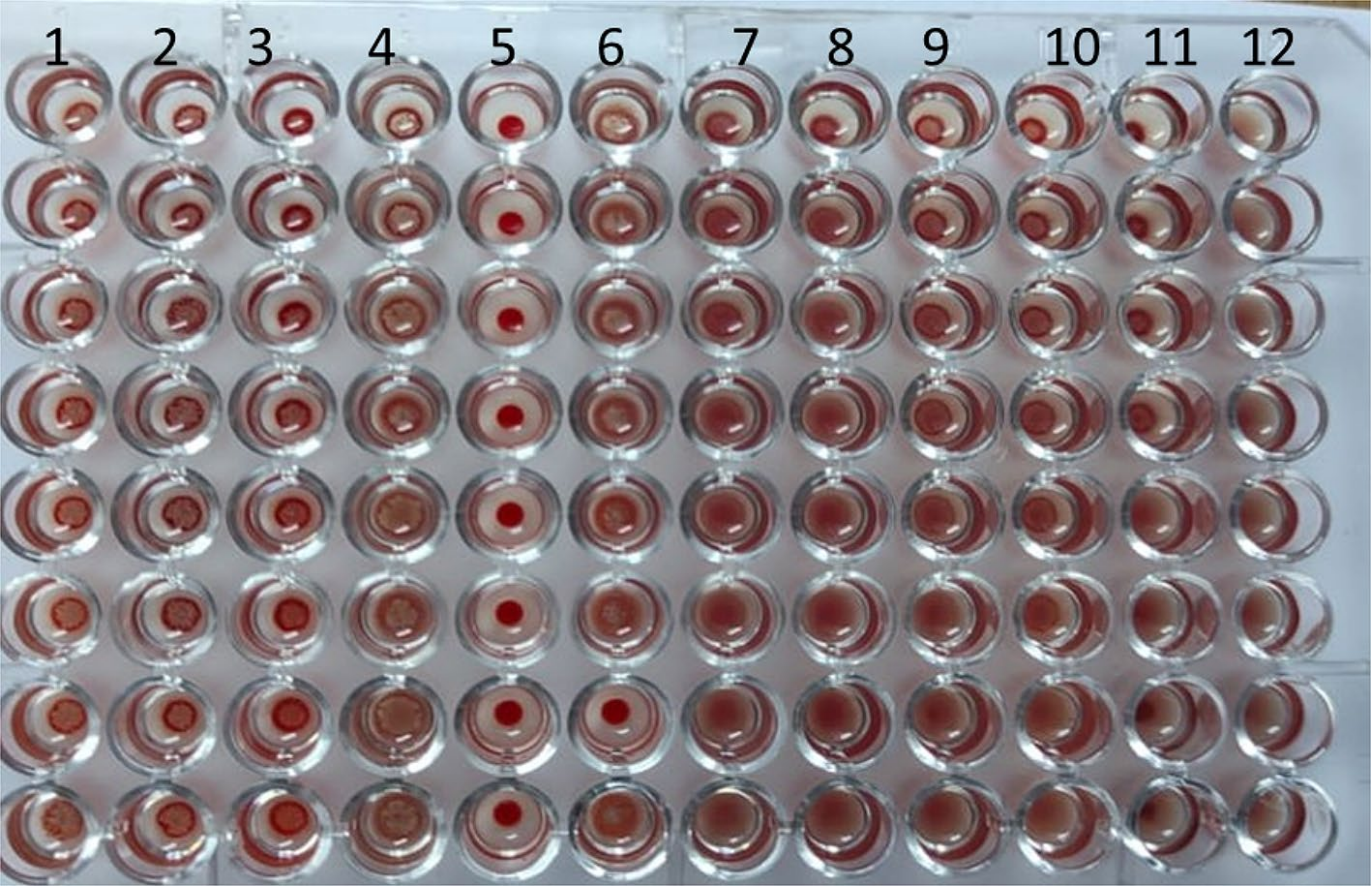
show the effect of different sugars on the inhibition of the haemagglutination activity of lectins of lentil and soyabean On human blood group (O). Where: raws 1 to 6 = the effect of sugars on soyabean lectin activity. raws 7 to12 = the effect of sugars on lentil lectin activity. Biochemical changes in zucchini plants treated with lentil lectin (pre and post inoculation).

The haemagglutination inhibition test was performed in the presence of different sugars, and the results in Table indicated that the haemagglutination inhibition of lectins of lentil and soyabean; the lectins were inhibited by fructose, glucose, galactose, mannose and lactose sugars at different concentrations but not inhibited by Fig. 7.

#### Protein and carbohydrate content

**Figure 8:**
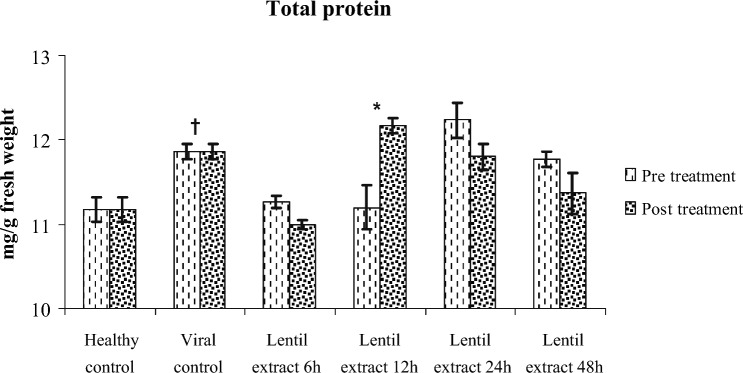
Effect of lentil extract on protein content in zucchini plants Pre and post virus inoculation at 6,12,24 and 48 h.The values are means of ten replicates ± standard error.

The results showed that the pre and post virus infection and treated with lentil lectin were significantly increased in total soluble protein content in zucchini leaves compared with healthy ones. The protein content was 11.00, 12.10, 11.80, 11.37 mg/g FW for pre virus inoculation at 6, 12, 24 and 48 h respectively. Also, the protein content was 11.27, 11.20, 12.20, 11.76 mg/g FW for post virus inoculation at 6, 12, 24 and 48 h respectively. Compared with healthy ones (control) 11.16 mg/g at 6 h the total protein almost equal to healthy ones Fig. 8. While total carbohydrate content was 26.17, 25.57, 25.00, 25.17 for pre-virus inoculation at 6, 12, 24 and 48 h respectively. Also, the Carbohydrates content was 26.40, 28.23, 25.36, 26.13 mg/g FW for post virus inoculation at 6, 12, 24 and 48 h respectively. In comparison to healthy ones, the total carbohydrate was increased at 6 h pre virus infection while increased at 6, 12 and 4 h post virus infection Compared with healthy ones (control) 25.93 mg/g.

#### Proline content

**Figure 9:**
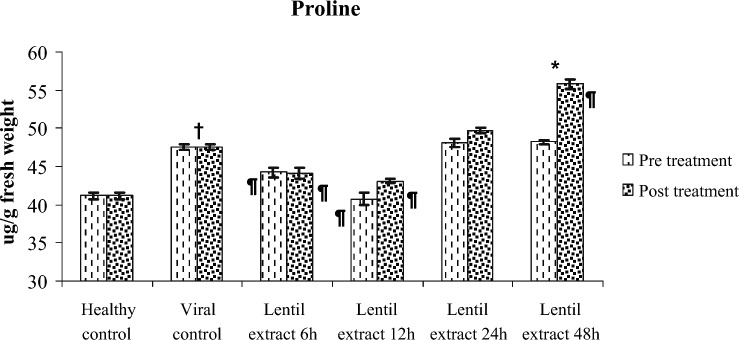
Effect of lentil extract on total chlorophylls in zucchini plants Pre and post virus inoculation at 6, 12, 24 and 48 h. The values are means of ten replicates ± standard error.

The proline content was 44.10, 43.06, 49.70, 50.80 ug/g for pre virus inoculation at 6, 12, 24 and 48 h respectively. Also, it is content was 44.20, 40.73, 48.04, 48.02 (ug/g FW) for post virus inoculation at 6, 12, 24 and 48 h respectively. Compared with healthy ones (control) (41.20 mg/g FW) total proline content is significantly increased in zucchini leaves compared with healthy ones Fig. 9. A 1.5-fold rise in proline content was also seen in leaves treated at 48 h in post treatment application.

#### The antioxidant enzymes activity (Peroxidase)

**Figure 10:**
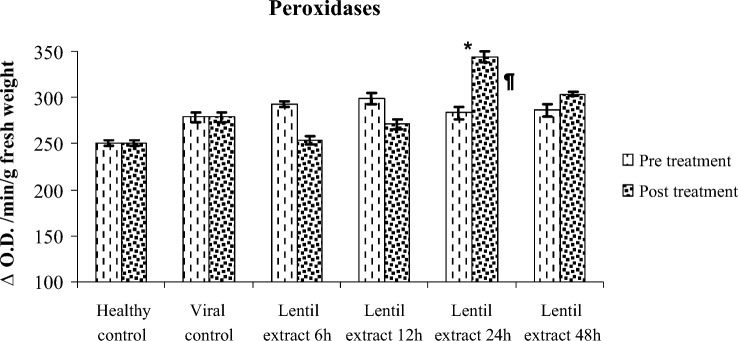
Effect of lentil extract on peroxidases in zucchini plants Pre and post virus inoculation at 6, 12, 24 and 48 h. The values are means of ten replicates ± standard error.

Results in Table 3 showed that significant increase of peroxidase enzyme activities in the pre and post virus infection and treated with lentil lectin. The peroxidase enzyme activity was 253.33, 271.00, 343.66, 304.00 (unit/g FW) for pre virus inoculation at 6, 12, 24 and 48 h respectively. Also, the peroxidase enzyme activity was 293.00, 299.00, 283.33, 286.00 (unit/g FW) for post virus inoculation at 6, 12, 24 and 48 h respectively compared with healthy ones (control) 250.00 (unit/g FW) Fig. 10.

**Table 3:** Molecular weight of detected bands.

MW	1	2	3	4	5	6	7	8	12	13
145	1	1	1	1	1	1	1	1	0	0
132	1	1	1	1	1	1	1	1	1	0
129	1	1	1	1	1	1	1	1	1	1
108	1	0	0	0	0	1	1	0	1	0
94	1	0	0	0	0	1	1	0	0	1
81	1	0	1	1	0	1	1	1	0	0
76	1	0	1	1	0	1	1	1	0	0
69	0	0	0	0	0	0	0	0	0	0
56	1	1	1	1	1	1	1	1	1	1
51	1	1	1	1	1	1	1	1	1	1
48	1	0	1	1	0	1	1	1	1	0
44	0	0	1	1	0	0	1	1	1	0
38	0	0	0	0	0	0	1	0	0	0
34	0	1	1	1	1	0	0	1	0	0
30	1	1	1	1	1	1	1	1	0	1
20	1	0	0	0	0	1	1	0	0	0
	12	7	11	11	7	12	14	11	7	5

#### Photosynthetic pigment content

**Figure 11:**
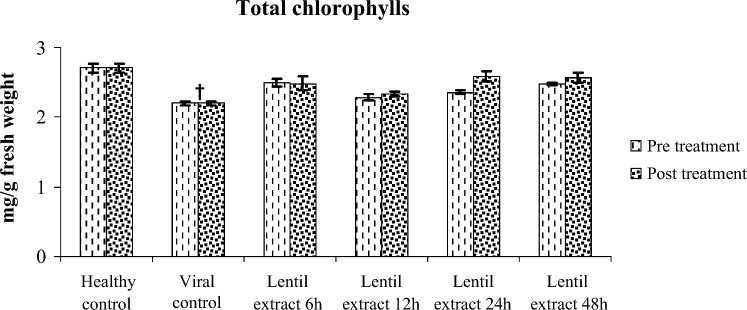
Effect of lentil extract on total chlorophylls in zucchini plants Pre and post virus inoculation at 6, 12, 24 and 48 h. The values are means of ten replicates ± standard error.

Pre and post virus infection and treated with lentil lectin were significantly decreased in total chlorophyll content in zucchini leaves compared with healthy ones. The chlorophyll content was 2.48, 2.33, 2.58, 2.57 mg/g FW for pre virus inoculation at 6, 12, 24 and 48 h respectively. Also, the chlorophyll content was 2.49, 2.28, 2.35, 2.48 mg/g FW for post virus inoculation at 6, 12, 24 and 48 h respectively. Compared with healthy ones (control) 2.71 mg/g Fig. 11.

#### Total phenols

**Figure 12:**
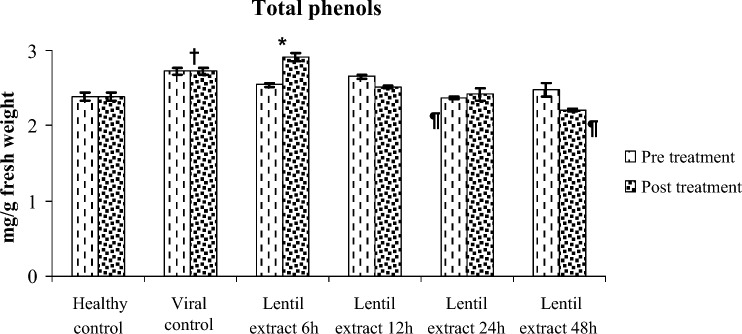
Effect of lentil extract on total phenols in zucchini plants Pre and post virus inoculation at 6, 12, 24 and 48 h. The values are means of ten replicates ± standard error.

The significant differences between pre and post treatment were in total protein in group treated by lentil 12 h;total phenols in group treated by lentil 6 h; proline in group treated by lentil 48 h; peroxidases in group treated by lentil 24 h; total carbohydrates in group treated by lentil 12 h; free amino acids in all treatd groups shown in Fig. 12.

#### Analysis of protein patterns of antiviral proteins in treaed leaves by polyacrylamide gel electrophoresis

Polyacrylamide gel electrophoresis was performed on the treated leaves by lentil lectin to see if any novel protein bands could by induced in treated leaves by the tested lectins.

**Figure 13:**
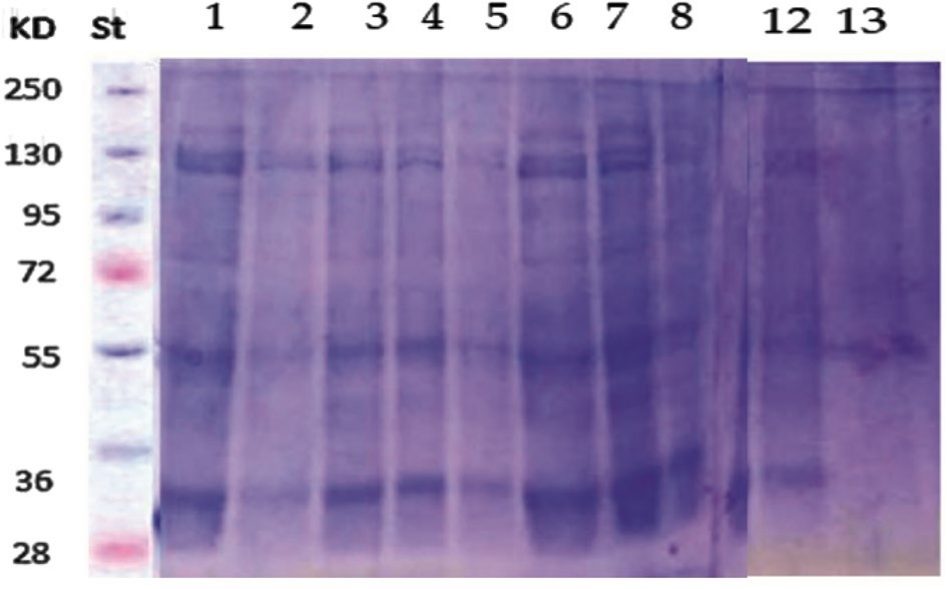
Electrophoresis of general protein patterns separated by using (SDS-PAGE) of healthy, infected and treated with plant lectin leaves. Lane St: standard protein. Lane 1: pre- inoculation by lentil extract at 6 h. Lane 2: pre- inoculation by lentil extract at 12 h. Lane 3: pre- inoculation by lentil extract at 24 h. Lane 4: pre- inoculation by lentil extract at 48 h. Lane 5: post- inoculation by lentil extract at 6 h. Lane 6: post- inoculation by lentil extract at 12 h. Lane 7: post- inoculation by lentil extract at 24 h. Lane 8: post- inoculation by lentil extract at 48 h. Lane 12: Healthy control. Lane 13: infected with ZYMV.

Ten treatments of zucchini plant by lentil lectin showed in (Fig. 13 and Table 3). Lane 12 presented untreated control and showed 7 bands, the bands (48–44 KDa) refer to 8S, phasolin bands, which disappear in infected plants in lane 13 that showed only five bands. On the other hand, the treated samples in lanes 1, 3, 4, 6, 7showed protein bands more than other samples i.e., 12, 11, 11, 12 and 14 bands respectively. 7S bands (76–72 KDa) appear in samples 1, 3, 4, 6 and 7 while 8S bands appear in 1, 3, 4, 6, and 7 samples, while 11S protein appear in samples 1, 3, 4, 6 and 7 and lectin bands appear in plant samples 1, 6, and 7. The SDS-PAGE showed that post treating zucchini plant with lentil extract for 24 h is the best treatment in exerting new protein in plant followed by samples 1, 6, and 3 in descending order.

## Discussions

Plant viral diseases are a major threat to many economic sustainable and productive crops worldwide, resulting in a significant financial loss every year^28^. It is challenging to eradicate or manage virus infections due to the intricate and dynamic structure of virus epidemics and the high rate of virus evolution^29^. New methods for developing viral resistance through biotic or abiotic inducers have been developed in an effort to provide a simple, long-lasting remedy. Two strategies are used in agriculture to manage disease: preventive measures to prevent virus spread and immunization to develop virus-resistant plants^30^. There have been many substances of natural and synthetic origin that exhibit an inhibitory effect against phytopathogenic viruses that have been studied, but none of them have a satisfactory selective action that allows them to be used for the specific prophylaxis and treatment of plant viral diseases^31^. In the present study, the potential activity of two plant lectins extracted from *L. culinaris* and Glycine max was assayed against ZYMV infection for their ability to protect C. pepo plants under greenhouse conditions. Application of the *L. culinaris* and Glycine max before ZYMV infection at various time intervals revealed that these lectins had potent antiviral activity against ZYMV, and produced notable inhibition at the first 6 h before ZYMV inoculation. The inhibitory action was slightly reduced when the inhibitor was applied at 12, 24, 36, 48, and 72 h before virus inoculation. The plant lectins treatment after ZYMV inoculation caused significant inhibition when applied at 1 h after viral inoculation, but less so at the other times (6, 12, 24, 48, and 72 h), which gave 393 significant inhibition. These results were similar to which reported by^9^. These results were similar to results reported by^11^. When 395 applying biotic inducers (M. jalapa, C. inerme, mixture and kombucha) pre- and post- virus 396 inoculation resulted in an increase the plant tolerant of infection. Also, these results were agreed 397 with^32^. Applications made prior to vaccination were found to be superior to vaccinations given afterward. Without influencing the host’s metabolism, the generated antiviral chemicals indirectly inhibit the replication process and the criteria of the symptoms established. Analysis of morphological and physiological data demonstrated that viral infections and *L. culinaris* and Glycine max lectin treatments affected metabolism. The presented data indicated that ZYMV—infection of C. pepo led to a high decrease in shoot length, root length, fresh weight, and number of flowering plants. This was in relation to the changes in morphological criteria caused by ZYMV infection and treatment by antiviral, produced from the *L. culinaris* and Glycine max lectins. In contrast, when each antiviral was combined with ZYMV, Zuchini plants’ fresh weight, shoot length, and root increased. These findings are in line with those of Galal, 1989 who discovered that plant extracts containing virus caused an increase in the morphological traits in squash plants inoculated with CMV in in vitro and in vivo tests. It is important to note that higher plant viruses disrupt many of their metabolic processes by reducing or boosting one or more crucial stages. In this regard, ZYMV-infected and treated *C. pepo* leaves were examined for alterations in a number of metabolic pathways. In the present investigation, results clearly appear a reduction 413 in the total chlorophyll in zucchini plant leaves due to ZYMV infection where^33^ reported that the appearance of systemic chlorosis due to virus infection is accompanied 415 by a decrease in photosynthesis. According to^34^ decreases of chlorophyll 416 were the result of the blocking Chl synthesis. On the other hand^35^) thought that it could be due to increased chlorophyllase activity. In the 418 analysis of our results, we found that ZYMV infection cause a significant decrease in total 419 chlorophyll content of infected leaves. Similar results were published by^36,37^ Also, a decrease in net photosynthetic rate often accompanied with a decrease in chlorophyll contents in virus infected plants. However, the results of treatments showed significantly decrease in ZYMV symptoms also the chlorophyll content increase in comparison to infected leaves. According to^35^, salicylic acid improved the carotenoid molecules’ resistance to oxidation, preventing the breakdown of other pigment ingredients. According to the results of this study, it was found to reduce ethylene production, stimulate the development of carotenoids, which shield chlorophyll from oxidation, and ultimately enhance chlorophyll concentration. The 428 total carbohydrate contents are dramatically reduced with ZYMV infection because the chlorophyll content decreases in response to ZYMV infection. Typically, infected leaves exhibit a buildup of starch and a reduction in the content of soluble carbohydrates^36,38^. Total carbohydrate content significantly rises after lectin treatments. The soyabean lectin for seed soaking at 4 h as well as post inoculation of lentil lectin for 12 h had the highest carbohydrate content scores. Total protein levels significantly increased in response to viral inoculation compared to the unvaccinated control^39,40^. All of the results point to significant metabolic alterations brought on by viral infections or *L. culinaris* extract treatments, and they also suggest that exogenous extract treatments are essential for enacting the plant's defences against ZYMV infection.

### Supplementary Information

Supplementary Figures.

## Data Availability

All data are included in this article and in the supplementary materials.

## Abbreviation

ZYMV: *Zucchini yellow mosaic virus*
