# Supplementary material for: Investigates the ability of plant extracts from Lens culinaris to protect zucchini from the Zucchini yellow mosaic virus (ZYMV)
Source: Sci Rep. 2024 May 28;14:12257. doi: 10.1038/s41598-024-62128-6 (PMC11133456; doi:10.1038/s41598-024-62128-6)
Supplement: Supplementary file 1 — Supplementary Figures. [file 41598_2024_62128_MOESM1_ESM.pdf]

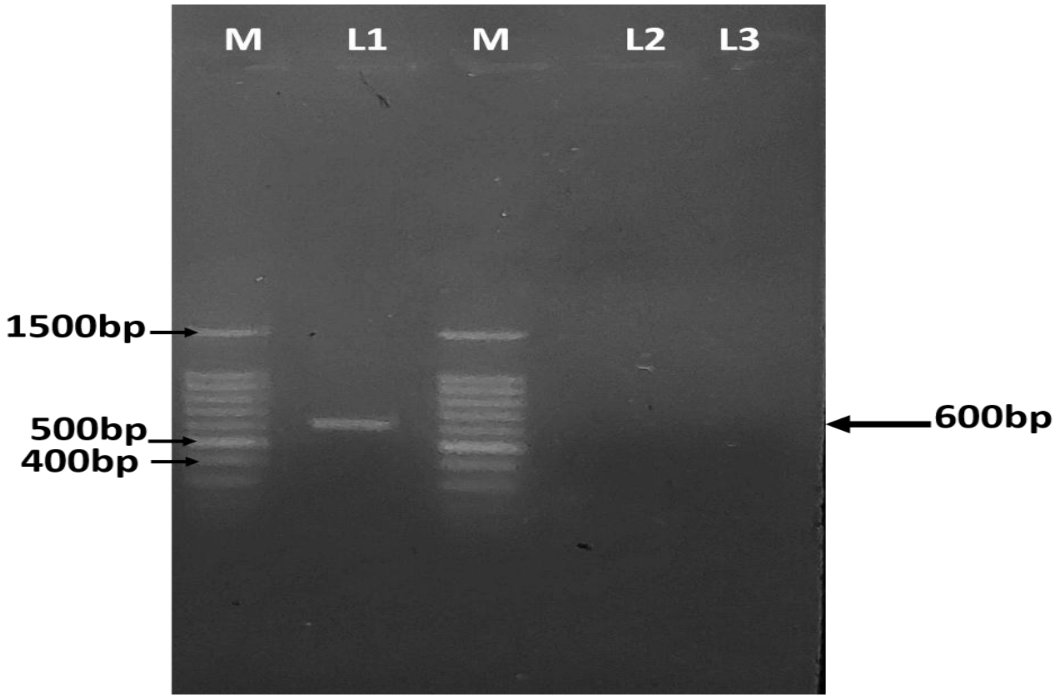

Supplementary Figure 1 : Agarose gel electrophoresis patterns of RT-PCR products of ZYMV isolate.

M: 100 bp DNA ladder (Biomatik).
L1: Sample of the naturally infected squash plant.
L2 and L3: Negative Squash plant (Healthy).

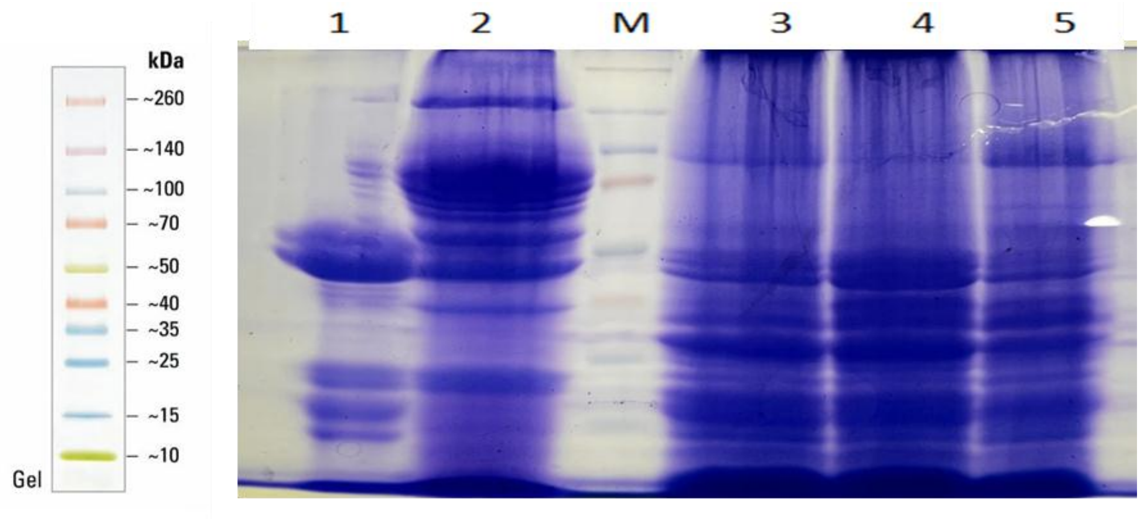

Supplementary Figure 2 : SDS-PAGE electropherogram showed the patterns of the purified lectins.
